# Cytotoxicity and Differentiating Effect of the Poly(ADP-Ribose) Polymerase Inhibitor Olaparib in Myelodysplastic Syndromes

**DOI:** 10.3390/cancers11091373

**Published:** 2019-09-16

**Authors:** Isabella Faraoni, Maria Irno Consalvo, Francesca Aloisio, Emiliano Fabiani, Manuela Giansanti, Francesca Di Cristino, Giulia Falconi, Lucio Tentori, Ambra Di Veroli, Paola Curzi, Luca Maurillo, Pasquale Niscola, Francesco Lo-Coco, Grazia Graziani, Maria Teresa Voso

**Affiliations:** 1 Pharmacology Section, Department of Systems Medicine, University of Rome Tor Vergata, 00133 Rome, Italy; 2 Hematology Section, Department of Biomedicine and Prevention, University of Rome Tor Vergata, 00133 Rome, Italy; 3 Hematology Unit, Tor Vergata Hospital, 00133 Rome, Italy; 4 Hematology Unit, “S. Eugenio” Hospital, 00144 Rome, Italy

**Keywords:** MDS, PARP inhibitors, olaparib, hematopoietic differentiation, PARP1, AML

## Abstract

Myelodysplastic syndromes (MDS) are highly heterogeneous myeloid diseases, characterized by frequent genetic/chromosomal aberrations. Olaparib is a potent, orally bioavailable poly(ADP-ribose) polymerase 1 (PARP1) inhibitor with acceptable toxicity profile, designed as targeted therapy for DNA repair defective tumors. Here, we investigated olaparib activity in primary cultures of bone marrow mononuclear cells collected from patients with MDS (*n* = 28). A single treatment with olaparib induced cytotoxic effects in most samples, with median IC_50_ of 5.4 µM (2.0–24.8 µM), lower than plasma peak concentration reached in vivo. In addition, olaparib induced DNA damage as shown by a high proportion of γH2AX positive cells in samples with low IC_50_s. Olaparib preferentially killed myeloid cells causing a significant reduction of blasts and promyelocytes, paralleled by an increase in metamyelocytes and mature granulocytes while sparing lymphocytes that are not part of the MDS clone. Consistently, flow cytometry analysis revealed a decrease of CD117+/CD123+ immature progenitors (*p* < 0.001) and induction of CD11b+/CD16+ (*p* < 0.001) and CD10+/CD15+ (*p* < 0.01) neutrophils. Morphological and immunophenotypic changes were associated with a dose-dependent increase of PU.1 and CEBPA transcription factors, which are drivers of granulocytic and monocytic differentiation. Moreover, the combination of olaparib with decitabine resulted in augmented cytotoxic and differentiating effects. Our data suggest that olaparib may have therapeutic potential in MDS patients.

## 1. Introduction

Myelodysplastic syndromes (MDS) are a group of highly heterogeneous diseases characterized by peripheral blood cytopenia, dysplasia in one or more hematopoietic cell lineages and a differentiation defect, with an increased risk of evolving to acute myeloid leukemia (AML) [1]. To date, only few treatment options are available in these diseases, including growth factors in lower-risk MDS and the hypomethylating agents decitabine and azacitidine in intermediate/high-risk MDS. Both agents delay progression to AML by exerting cytotoxic and differentiating effects. Moreover, azacitidine has been shown to prolong overall survival [2]. However, especially in patients with higher-risk MDS, ineligible for allogeneic stem cell transplantation, life expectancy remains dismal. Therefore, innovative treatment strategies are warranted for elderly and unfit patients.

Poly(ADP-ribose) polymerase inhibitors (PARPi) are compounds with a favorable tolerability profile, designed as targeted therapy for homologous recombination (HR)-defective tumors. The FDA and EMA have approved some PARPi as maintenance therapy for platinum-sensitive relapsed (olaparib, rucaparib, and niraparib) or newly diagnosed (olaparib) high-grade epithelial ovarian, fallopian tube or primary peritoneal cancers. Olaparib and rucaparib received market authorization also for the treatment of relapsed BRCA1 or BRCA2 mutated advanced ovarian cancer, after previous lines of chemotherapy and independently of platinum sensitivity [3]. Recently, olaparib and talazoparib were FDA approved for patients with epidermal growth factor receptor 2 (HER2)-negative metastatic breast cancer with BRCA mutations, relapsing after previous chemotherapy. These and other PARPi are also under clinical development as monotherapy or in combination with targeted agents or chemotherapy for different types of cancer. In fact, accumulating evidence suggests a potential therapeutic role for PARPi in tumors characterized by mutations in genes involved in the HR repair of DNA double strand breaks (DSBs), including RAD51, ATM, ATR, CHEK1/2, FANC family members [4,5] (reviewed by Gadducci and Guerrieri [6]), or HR upstream modulators as PTEN [7] or IDH1 and IDH2 [8] (reviewed by Nickoloff et al. [9]). Therefore, regardless of BRCA mutational status, other defects in DNA repair may induce a “BRCAness” phenotype that would increase tumor sensitivity to PARPi.

We and others recently demonstrated that olaparib and other PARPi exert anti-proliferative and proapoptotic effects in human AML blasts at clinically achievable concentrations [10,11,12,13,14,15,16,17,18] that do not affect the viability of normal bone marrow (BM) stem cells [10,12,18]. The low BRCA1/2 RNA and protein expression levels detected in AML blasts [10] and myeloproliferative neoplasms [19] might account for myeloid malignancies sensitivity to synthetic lethality induced by PARPi. Also, some chromosomal translocations which are recurrent in leukemia including t(8;21) (RUNX1-RUNX1T1), t(15;17) (PML-RARA) [12] and t(17;19) (TCF3-HLF) [14] can weaken the HR repair activity and sensitize AML cells to PARPi treatment (reviewed by Faraoni et al. [20]).

Besides its involvement in DNA repair, through post-translational protein modifications (PARylation), the founding member of the PARP family PARP1 has a crucial role in the regulation of chromatin structure and gene transcription [21,22,23] (reviewed by Hottiger [24]). Recently, we suggested that olaparib cytotoxicity on primary AML blasts is the result of drug-induced DNA damage as well as of alternative mechanisms, such as upregulation of death receptor RNAs and proteins (FAS, DR5 and DR4), which in turn requires NF-κB activation [16]. The involvement of PARP1 in modulation of gene expression suggests additional therapeutic implications of PARPi in hematological diseases. In particular, deregulated gene expression in hematopoietic stem cells is regarded as a hallmark of MDS, with involvement of different pathways depending on the disease stage [25]. Interestingly, higher-risk MDS are characterized by a decrease of apoptosis and high levels of genomic instability, likely as a consequence of the observed alterations in DNA damage response pathways [25,26].

On this basis, we investigated the effects of PARP1 inhibition in primary cultures of BM mononuclear cells (BM-MNC) collected from MDS patients. Our results indicate that olaparib is not only cytotoxic, but importantly, also stimulates differentiation of immature MDS myeloid cells, as single agent or in combination with decitabine.

## 2. Results

### 2.1. MDS Mononuclear Cells Are Sensitive to Olaparib In Vitro

To assess the sensitivity of MDS to olaparib, experiments were performed using short-term cultures of MNC freshly isolated from the BM samples of 28 MDS patients. Table 1 lists the patients’ characteristics.

**Figure 1:**
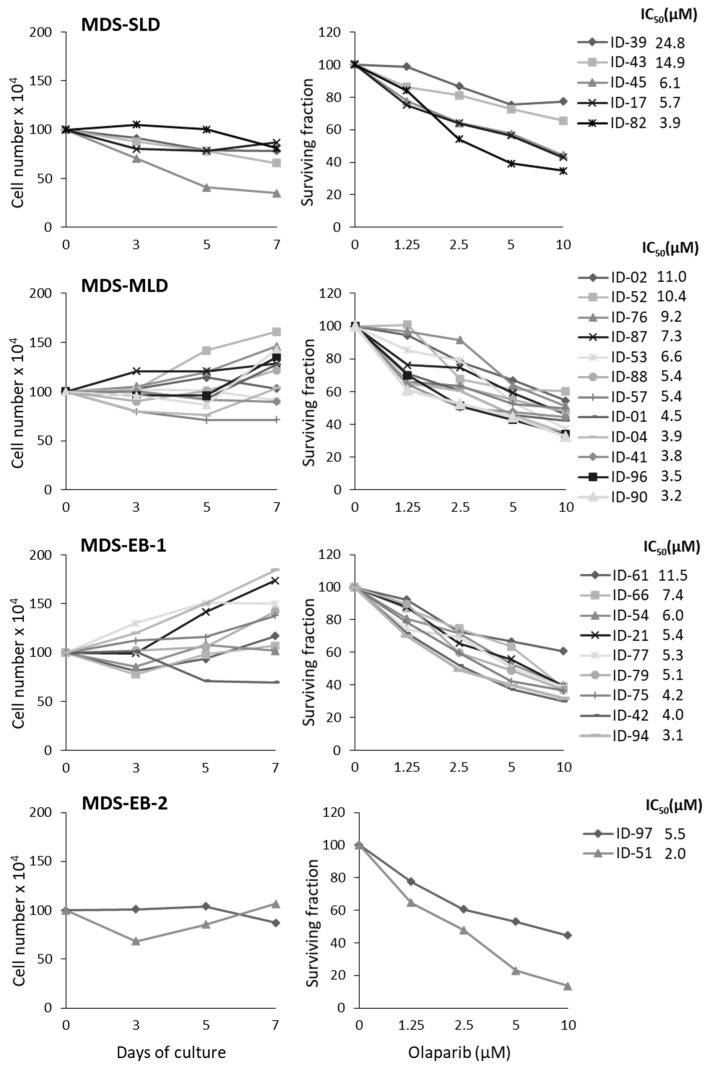
Olaparib exerts cytotoxic effects in primary MDS cultures. BM-MNC collected from MDS patients were cultured with IL-3, SCF and FLT3LG and treated (time 0) with increasing concentrations of olaparib. For each primary culture, cell proliferation was evaluated by counting viable cells using trypan blue exclusion at 3, 5 and 7 days. Standard deviation (SD) of four replicate counts was ≤20% and is not shown in the figure. MDS samples were grouped according to morphology, as MDS-SLD, MDS-MLD, MDS-EB-1 and MDS-EB-2. Left graphs represent the proliferation pattern of untreated primary MDS cells during 7 days of culture. Right graphs show the surviving fractions after 7 days of treatment, and the olaparib IC_50_s for each sample calculated with respect to untreated cells cultured for the same time period.

The proliferation rate of untreated or olaparib-treated primary cells was monitored for 7 days and the results are shown by grouping samples according to the 2016 WHO classification of MDS [1]. Some of the untreated MDS cultures showed a slight increase in cell number (*n* = 12), while the remaining samples either did not proliferate (*n* = 7) or showed a reduction in cellularity (*n* = 9) (Figure 1, left panels). Nevertheless, viability was constantly ≥70% in control cells during the culture. No significant correlation was found between the cell growth in culture and MDS risk according to the Revised International Prognostic Scoring Systems (R-IPSS). Treatment with olaparib induced a dose-dependent decrease of cell survival in all MDS samples with a median IC_50_ of 5.5 µM (range 2.0–24.8 µM) (Figure 1, right panels). The median IC_50_ values were comparable in the four MDS subgroups analyzed (MDS-SLD, 6.1 µM; MDS-MLD, 5.4 µM; MDS-EB-1, 5.3 µM; MDS-EB-2, 3.8 µM). No statistically significant correlation was detected between the proliferation indexes of MDS cultures and olaparib IC_50_s, suggesting that the drug sensitivity did not depend on cell ability to grow in vitro. Representative growth curves of olaparib-treated samples with comparable olaparib IC_50_ values but different proliferation rates are shown in Figure S1A. Also, we found no significant correlation between cell sensitivity, expressed as olaparib IC_50_ values at 7 days, and the MDS prognostic variables listed in Table 1. Notably, the olaparib IC_50_ values were in most cases largely below the steady-state plasma peak concentrations (C_max_ = 16–22 µM), measurable in patients with solid tumors receiving 300 mg olaparib twice daily [27,28]. Conversely, olaparib sensitivity of normal BM, CD34-enriched mobilized peripheral blood and purified CD34+ samples (IC_50_ range: 18.5–27.0 µM) was markedly higher than that of MDS cells (Figure S1B), in agreement with previous findings [5,10,12,29].

**Figure 2:**
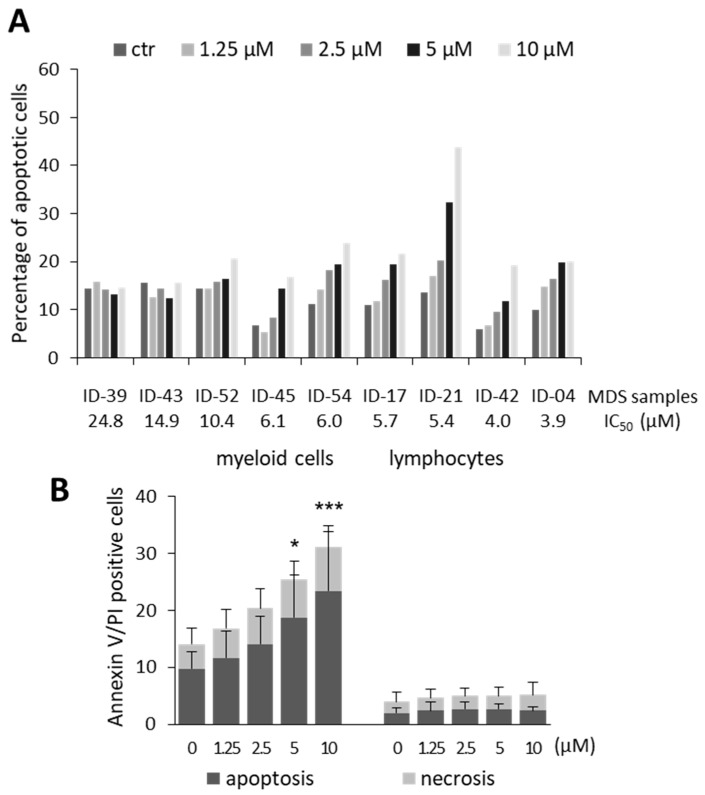

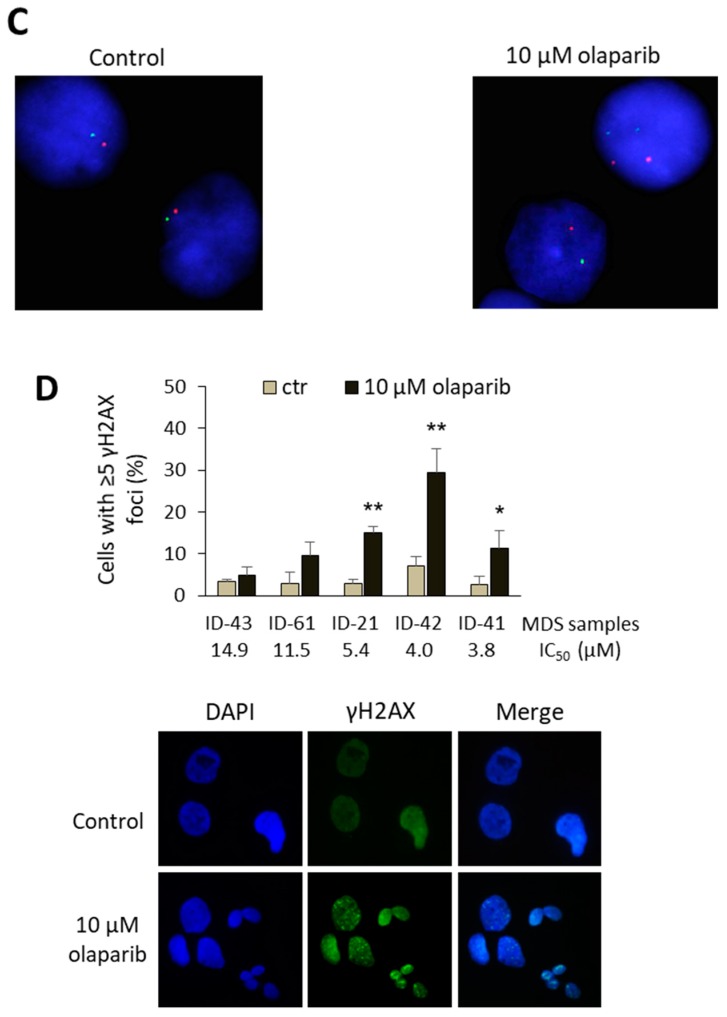
Apoptosis and DNA damage in primary MDS in vitro cultures treated with olaparib. (**A**) Percentages of apoptotic cells (Annexin V positive/PI negative), detected by FACS analysis, in gated myeloid cells after exposure to graded concentrations of olaparib (7 days) compared to untreated cultured cells. Samples were classified in order of decreasing olaparib IC_50_ values. (**B**) Histograms indicate the mean percentage ±SD of apoptotic and necrotic cells (Annexin V positive/PI positive), detected within the myeloid and lymphocytic populations, respectively. Data refer to six MDS samples after exposure to graded concentrations of olaparib for 7 days compared to untreated cultured cells. Olaparib induces apoptosis in the myeloid MDS cell compartment, while sparing lymphocytes. Statistical analysis was performed on apoptotic cells (Annexin V positive/PI negative cells) by Friedman followed by Dunn’s test. (**C**) Interphase FISH analysis of representative MDS cells with 7q deletion (ID-75). Images were taken at 100× magnification. Nuclei with deletions involving both critical regions at 7q22 and 7q36 show one red and one green signal only, whereas cytogenetically normal cells show two red and two green signals. In the control group, 75% of cells showed the 7q deletion, whereas in the olaparib-treated group this percentage dropped to 50%. (**D**) Analysis of γH2AX foci by immunofluorescence after 72 h of exposure to 0 and 10 μM olaparib. Values are the mean percentage ± SD of the cells with ≥5 γH2AX foci in four quadrants, containing at least 50 cells each. Statistical analysis was performed by Mann-Whitney test. Representative nuclei of MDS cells untreated or exposed to 10 μM olaparib are shown. Cells were probed with γH2AX (green stain) and DAPI (blue). Images were taken at 40× magnification. * *p* <0.05, ** *p* <0.01, *** *p* <0.001 vs. untreated control.

We then investigated whether the growth inhibitory activity of olaparib in MDS cells was associated with cytotoxic effects. Apoptosis was evaluated by cell staining with annexin V/PI and FACS analysis after 7 days exposure to graded concentrations of olaparib. Cells were gated in order to separately analyze apoptosis induction within the myeloid and lymphocyte populations. Dose-dependent apoptosis was observed in the myeloid compartment of MDS samples characterized by IC_50_ values ≤ 6.1 µM (Figure 2A), without major differences among cells at different maturation stages. Pooled statistical analysis of data referring to samples with olaparib IC_50_ values ≤ 6.1 µM indicated a significant increase in the percentage of apoptotic cells at 5–10 µM olaparib concentrations (Figure 2B). On the other hand, a negligible percentage of apoptotic cells was detected in the lymphocyte population (Figure 2B). Representative plots in Figure S2A demonstrate the lack of annexin V/PI staining in CD45-positive and CD33-negative lymphocytes. Lack of apoptosis detection in the lymphocyte population was not due to a faster killing kinetics in lymphoid cells, since apoptosis induction was not observed at an earlier time point (i.e., 3 days) (Figure S2B). These data suggest that olaparib preferentially kills myeloid precursors, but spares lymphocytes that are not part of the MDS clone. To further defining the targets of olaparib cytotoxic effects, two MDS samples with cytogenetic abnormalities were FISH analyzed after 7 days exposure to olaparib. The PARPi induced a 22% and 34% decrease in the number of cells with trisomy 8 and 7q deletion, respectively. Figure 2C shows representative FISH images of MDS cells with deleted chromosome 7. These data suggest a preferential killing of malignant cells by olaparib.

We then assessed whether the cytotoxic effects of olaparib in MDS might be attributable to DNA damage, using immunofluorescence analysis of γH2AX foci, which are markers of DSBs. The histogram in Figure 2D represents the percentage of γH2AX positive cells in five MDS samples untreated or treated with 10 μM olaparib for 72 h. The results indicate that the PARPi induced a significantly higher percentage of γH2AX positive cells in samples characterized by low IC_50_s (ID-21, ID-42 and ID-41), as compared to the untreated control. Conversely, a negligible increase in DNA damage was observed in two other MDS samples (ID-43 and ID-61) that were more resistant to the PARPi. These data indicate that treatment with single agent olaparib induces DNA damage that accounts, at least in part, for olaparib cytotoxicity.

### 2.2. Differentiating Effects of Olaparib on MDS Immature Myeloid Cells

**Figure 3:**
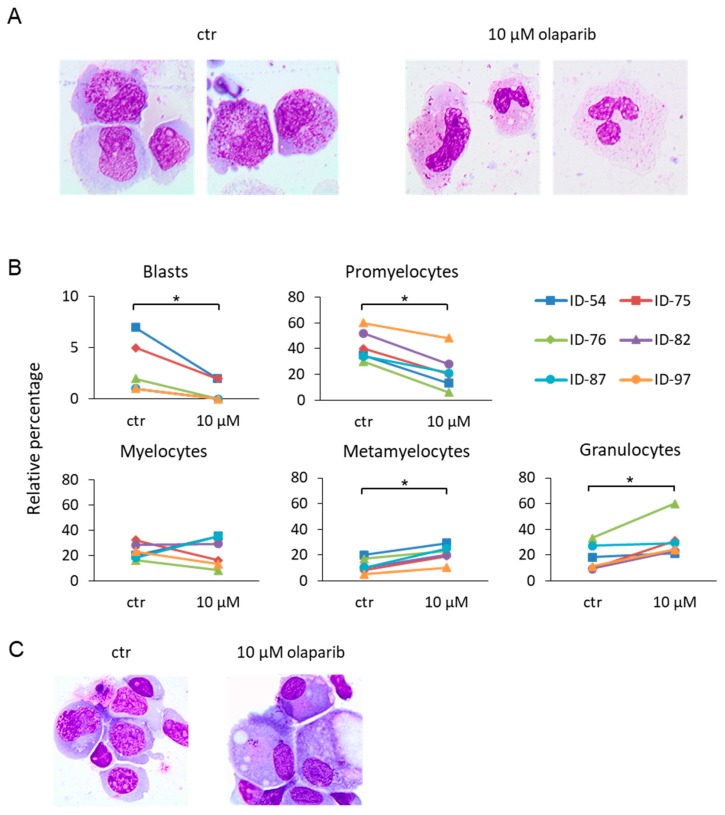
Morphological analysis of MDS samples treated with olaparib. May Grunwald-Giemsa staining of cytospins from six primary MDS samples after 7 days of culture. Untreated cultured cells and those treated with 10 μM olaparib are shown. (**A**) Representative pictures are shown (sample ID-54). Images were taken at 100× magnification. (**B**) Differential counts using standard microscopy were performed by two specialized hematologists (ADV and MTV). Statistical analysis by paired Wilcoxon test: * *p* < 0.05. (**C**) Sample ID-79 after 7 days of 10 μM olaparib treatment. Images were taken at 100× magnification.

Flow cytometry analysis by side scatter (SSC) vs forward scatter (FSC) and SSC vs CD45 showed an increase in the mature cells (high-SSC) after treatment with olaparib (Figure S2A, left and middle panels), suggesting a potential PARPi effect on myeloid cell differentiation. Based on these findings, we studied morphological changes in MDS samples upon treatment with 10 µM olaparib for 7 days, after May Grunwald-Giemsa staining of cytospins. We observed a significant reduction of the proportion of blasts and promyelocytes, paralleled by an increase of metamyelocytes and mature granulocytes (Figure 3A,B). In cells from one case of MDS-EB-1, olaparib treatment induced outgrowth of 22% monolobulated megakaryocytes, as depicted in Figure 3C.

**Figure 4:**
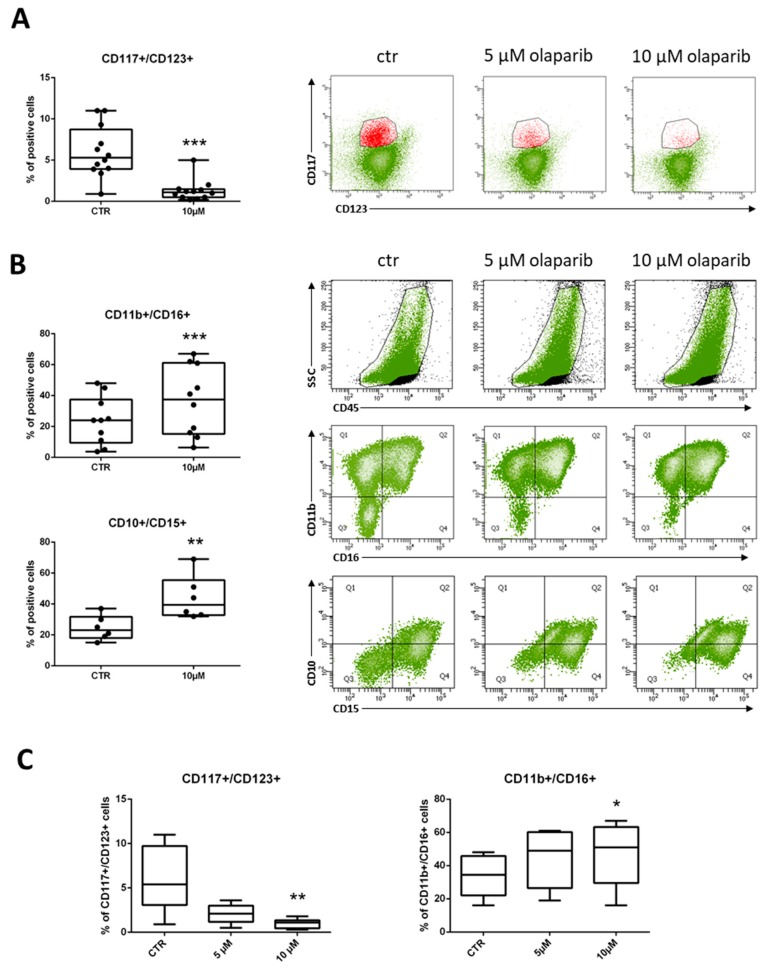
Immunophenotypic analysis of myeloid cells in MDS samples treated with olaparib. (**A**) Flow cytometry analysis of MDS samples treated with olaparib for 7 days vs untreated control cells cultured for the same time period. Box-whisker plots show the proportion of CD117+/CD123+ immature progenitors in MDS samples (*n* = 13; paired Wilcoxon test: *** *p* <0.001). Representative plots show the changes in the expression of CD117+/CD123+ markers (red) in sample ID-76 after treatment with 5 or 10 µM olaparib. (**B**) Box-whisker plots show the proportion of CD11b+/CD16+ (*n* = 10) and CD10+/CD15+ (*n* = 6) mature neutrophils in MDS samples (paired Wilcoxon test: ** *p* < 0.01, *** *p* < 0.001). Representative plots of flow cytometry analysis of CD45+ cells, after lymphocyte (black) exclusion, showing changes in SSC and increased expression of CD11b+/CD16+ or CD10+/CD15+ cells in ID-76 sample after treatment with 5 or 10 µM olaparib. (**C**) Box-whisker plots showing the olaparib dose-dependent effect in CD117+/CD123+ (left panel) and CD11b+/CD16+ (right panel) cells from six samples treated with 5 or 10 µM olaparib. Statistical analysis by Friedman followed by Dunn’s test: * *p <* 0.05, ** *p* < 0.01.

Differentiation was also analyzed by means of CD117 and CD123 antigen expression, which are markers of myeloid progenitors. The percentage of CD117+/CD123+ immature progenitors was significantly reduced in treated cells, as compared to untreated controls in 12 of 13 MDS samples (Figure 4A). Representative dot-plots of the olaparib-induced decrease in CD117+/CD123+ cell population are shown (Figure 4A, right panels). The lack of CD34 expression is known to be a common aberrancy in MDS immature myeloid compartment [30]. Indeed, the percentages of CD34+ stem cells were scored ≥1% in four of 12 samples only. Treatment with olaparib resulted in a marked reduction of CD34+ cells in three out of four samples, with no effects observed in the ID-02 sample that was the most resistant to the toxic effects of olaparib (Figure S3). Looking at neutrophil maturation patterns, samples treated with olaparib showed a significant induction of CD11b+/CD16+ cells (9 of 10) and CD10+/CD15+ (6 of 6) double positive cells, indicating differentiation towards mature neutrophils (Figure 4B). Representative dot-plots show a dose-dependent increase of CD45-positive/high SSC cells in the samples exposed to the PARPi. This effect was accompanied by a shift of the double-negative immature cells towards the double-positive mature population (Figure 4B).

In six MDS samples, we also tested whether modulation of differentiation markers was dose-dependent. Olaparib induced a reduction of CD117+/CD123+ MDS cells together with an increase of mature CD11b+/CD16+ cells that reached statistically significance at the concentration of 10 µM (Figure 4C). These data strongly suggest that inhibition of PARP1 can stimulate maturation of MDS myeloid precursors into neutrophils. A similar differentiating effect was also observed in one sample from a patient with low-blast count AML (Figure S4), which was also highly susceptible to olaparib cytotoxic effects (IC_50_ = 1.6 µM).

### 2.3. Olaparib Modulates the Expression of PU.1 and CEBPA Transcription Factors

**Figure 5:**
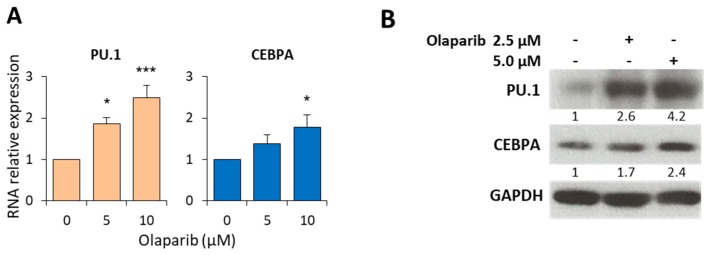
Olaparib modulates the expression of transcription factors associated with myeloid differentiation. (**A**) PU.1 and CEBPA mRNA expression were evaluated by qRT-PCR after 7 days of culture and treatment with 5 or 10 µM olaparib. Histograms show the mean ±SD relative expression in six MDS samples. Statistical analysis by Kruskal-Wallis followed by Dunn’s test: * *p* < 0.05, *** *p* < 0.001. (**B**) Representative immunoblot showing PU.1 and CEBPA protein expression from one MDS sample exposed to the indicated concentrations of olaparib (i.e., ID-54) for 7 days. Numbers below blots refer to the densitometric analysis of the immunoreactive bands and represent the fold-increase of protein expression normalized to GAPDH.

To confirm the ability of olaparib to stimulate myeloid differentiation, we studied the expression of the lineage-specific transcription factors PU.1 and CEBPA, drivers of granulocytic and monocytic differentiation. BM-MNC from six MDS patients were exposed to olaparib (5 and 10 µM), and total RNA analyzed by qRT-PCR after 7 days of treatment. We found that olaparib induced a statistically significant and dose-dependent upregulation of PU.1 RNA expression, while CEBPA was significantly upregulated at the 10 µM concentration (Figure 5A). This was associated with a dose-dependent increase of the corresponding proteins, as assessed by western blot analysis (Figure 5B).

### 2.4. Cytotoxic and Differentiating Effects of Olaparib and Decitabine Combined Treatment

To investigate the possible synergism between olaparib and decitabine, we initially used the OCI-AML2 leukemia cell line as a model. OCI-AML2 cells were treated with increasing concentrations of both drugs as single agents or in combination. After 3 days of culture, cells were analyzed by the MTS assay. Olaparib (1 to 8 µM) and decitabine (0.1 to 0.8 µM) exerted synergistic cytotoxicity on OCI-AML2 cells as assessed by the CompuSyn model [31] at all concentration ratios (Figure S5).

**Figure 6:**
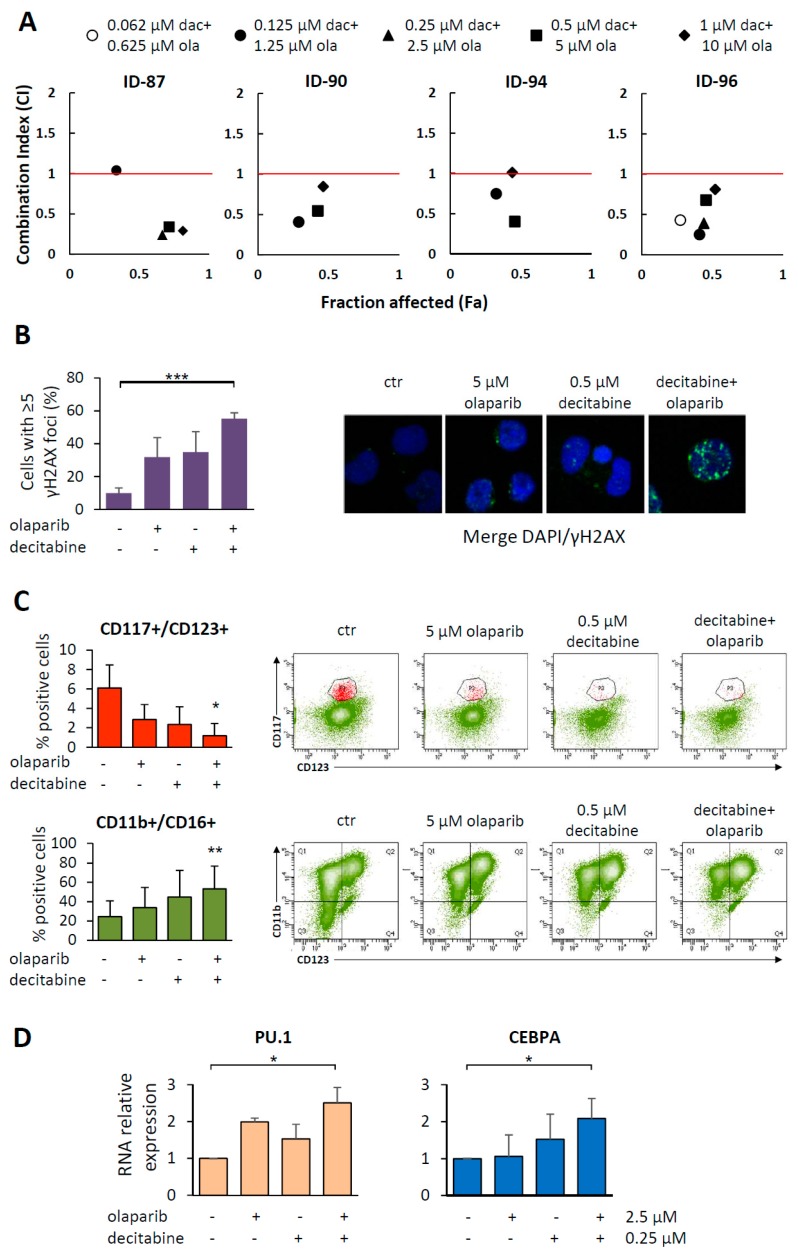
The olaparib and decitabine combination impairs survival and induces differentiation in primary MDS. (**A**) Primary MDS cells were treated with olaparib (ola, 0–10 µM) or decitabine (dac, 0–1 µM) or with different drug combinations at time 0. After 7 days of culture, cells were analyzed by the MTS assay to study cytotoxicity. Plots show the fraction of affected cells (Fa, *x* axis) and the combination index (CI, *y* axis) values as assessed by the CompuSyn model. Each plot indicates the CI values of a MDS samples treated at decitabine and olaparib 1:10 ratio. CI < 1, synergistic activity; CI = 1, additive effect; CI > 1, antagonism. (**B**) Analysis of γH2AX foci by immunofluorescence after 72 h of exposure of ID-90 cells to 5 μM olaparib and 0.5 µM decitabine, as single agents or in combination. Values are the mean percentage ±SD of the cells with ≥5 γH2AX foci in four quadrants, containing at least 50 cells each. Statistical analysis was performed by Kruskal-Wallis followed by Dunn’s test. Representative nuclei of untreated or drug-treated MDS cells are shown. Cells were probed with γH2AX (green stain) and DAPI (blue). Images were taken at 40× magnification. (**C**) Histograms show the mean percentage ± SD of CD117+/CD123+ and CD11b+/CD16+ cells in 3 MDS samples untreated or treated with 5 µM olaparib and 0.5 µM decitabine, as single agents or in combination. Statistical analysis by Friedman followed by Dunn’s test: * *p* < 0.05, ** *p* < 0.01. Representative dot plots showing data from a MDS sample (ID-88) untreated or treated with olaparib, decitabine or both drugs. A decrease of CD117+/CD123+ immature progenitors (upper plots) and increase of CD11b+/CD16+ (lower plots) are observed in treated samples as compared to the untreated control. (**D**) PU.1 and CEBPA mRNA expression evaluated by qRT-PCR after 7 days of treatment with olaparib and decitabine, as single agents or in combination. Histograms show the mean ±SD relative expression in three MDS samples. Statistical analysis by Kruskal-Wallis followed by Dunn’s test: * *p* < 0.05, ** *p* < 0.01, *** *p* < 0.001.

To assess the susceptibility to decitabine and olaparib combination (1:10), four MDS samples were exposed to equitoxic drug concentrations, including drug IC_50_ values and cytotoxicity was assayed by MTS after 7 days of treatment. In all samples, the two drugs exhibited synergistic cytotoxic effects at most of the concentrations tested (Figure 6A). In particular, for all samples the combination index (CI) values were <1 at the effective dose 50 (ED_50_). Moreover, analysis of the dose reduction index (DRI) indicated that addition of the PARPi to decitabine allowed 3.5–20.8 fold decrease of decitabine dose at 0.5 fraction affected (Fa). The highest DRI value was observed in ID-90 cells that exhibited the lowest sensitivity to decitabine. Cytotoxicity was associated with DNA damage, as indicated by the statistically significant increase of γH2AX positive cells in co-treated samples as compared to the untreated controls (Figure 6B).

To investigate whether the synergistic activity of decitabine plus olaparib did also impact on myeloid differentiation, we evaluated by FACS analysis the percentage of CD117+/CD123+ hematopoietic progenitors and CD11b+/CD16+ neutrophils in four MDS samples, untreated or treated with the indicated concentrations of the drugs. We found that the 0.5 µM decitabine and 5 µM olaparib combination significantly decreased the proportion of immature progenitors and increased the percentage of neutrophils (Figure 6C). The expression of PU.1 and CEBPA RNA was studied in three MDS samples and at least additive effects were observed in samples treated with the drug combination (Figure 6D).

## 3. Discussion

In the present study, we demonstrate for the first time that the PARPi olaparib used at clinically achievable concentrations [27,28] exerts cytotoxic and differentiating effects in short-term primary cultures of BM-MNC obtained from MDS patients.

The apoptotic effects of olaparib were observed both in actively proliferating and resting MDS cultures. These findings support the activity of PARPi also on quiescent malignant stem cells [10,15,16], that are generally more resistant to cytotoxic treatments. As recently suggested, the inefficient HR repair of DSBs in non-proliferating cells characterized by a “BRCAness” phenotype, does not account for the observed PARPi-mediated synthetic lethality, suggesting the involvement of additional repair mechanisms [15].

Interestingly, in our model, olaparib mainly targeted myeloid cells including the dysplastic cell lineages, while it spared lymphocytes, a cell subset that is not involved in the MDS clone. Consistently, normal hematopoietic cells showed lower sensitivity to olaparib as compared to MDS cells. This may indicate that PARPi-induced synthetic lethality targets MDS cells that are frequently characterized by DNA damage response defects which contribute to chromosome alterations involved in disease progression [25,32].

In our study, olaparib induced MDS cell differentiation, as indicated by the results of morphological and immunophenotypic analysis, and increased the expression of myeloid-specific transcription factors. In fact, the PARPi caused a reduction of CD117+ or CD34+ hematopoietic progenitors and an increase in maturing CD11b+/CD16+ and CD10+/CD15+ cells in almost all MDS analyzed samples. Since BM samples isolated from patients contain a mixture of normal and diseased cells with differentiation block, the observed increase in neutrophils might derive either from killing of immature MDS cells and consequent relative increase of healthy cells and/or from maturation of MDS hematopoietic progenitors. Actually, we found that olaparib-differentiated cells, as assessed by morphology, were still characterized by significant dysplastic changes, and the observed decrease of trisomy 8 and del(7)–positive cells in the two FISH-analyzed MDS samples was moderate. These data argue in favor to the hypothesis that the increase of differentiated cells in olaparib-treated cultures involves malignant cells and is not merely due to the killing of the latter and recovery of normal hematopoietic progenitors.

The regulatory effect of transcription factors determines the fate of hematopoietic stem cells from self-renewal towards generation of mature blood cells [33,34,35]. In our study, olaparib upregulated RNA and protein expression of the granulocytic and monocytic transcription factors PU.1 and CEBPA, both proteins reported to be frequently downregulated in myeloid malignancies [33,34,35]. The modulation of transcription factors involved in myeloid cell differentiation induced by olaparib might depend on the known ability of PARP1 to affect chromatin structure and RNA transcription by PARylation or direct interaction with target proteins [24,36,37]. In fact, PARP1 overexpression has been found to block differentiation in response to all-trans retinoic acid (ATRA) in leukemia cell lines [38] and a reduction of PARP1 expression was observed during differentiation of neutrophils or monocytes [39,40]. Interestingly, higher PARP1 RNA levels were observed in higher-risk MDS and correlated with inferior survival [41]. However, we cannot exclude that transcription factor modulation might be the consequence, rather than the cause, of differentiation induced by excessive DNA damage, as previously described in murine models of hematopoietic stem cells [42,43].

One clinical concern with olaparib is the risk of therapy-related MDS or AML associated to high-doses [44]. This risk has not been confirmed by a recently published phase 3 clinical trial which reported the occurrence of MDS or AML in 2% of olaparib-treated (300 mg, twice daily) vs 4% of placebo-treated patients [45]. Similar results were reported for the PARPi niraparib and rucaparib [46,47], suggesting that the burden of cytotoxic treatment prior to PARPi may be responsible for the development of therapy-related myeloid neoplasms.

In our study, the cytotoxic effects of the olaparib/decitabine combination were synergistic or at least additive in the MDS cell cultures. These findings are in line with the recently reported synthetic lethality induced by the association of the two drugs in a panel of AML cell lines [48,49]. Muvarak et al. demonstrated that low dose decitabine or azacitidine plus the PARPi talazoparib had synergistic cytotoxic effects in AML cell lines, as a consequence of increased DNA damage and delayed DNA repair [13]. In keeping with these results, the combination of the PARPi with a hypomethylating agent caused a marked antitumor response in an AML xenograft model [13]. The synergistic activity of PARPi with decitabine may also be explained by the involvement of PARP1 in the repair of decitabine-induced DNA lesions represented by randomly incorporated 5-aza-dC and trapped DNA methyltransferase 1 (DNMT1). In the presence of PARPi, DNA repair is impaired as shown by an increase of DBSs [13,48,49]. Noteworthy, the combination of olaparib and decitabine significantly decreased the proportion of immature myeloid progenitors and increased that of differentiated neutrophils. This effect was paralleled by the up-regulation of PU.1 and CEBPA transcription factors. The inhibition of DNA methylation caused by decitabine likely contributes to enhance the differentiating effect induced by olaparib. These results are of particular interest, considering that decitabine is approved by FDA for all subtypes of MDS and low-blast count AML (blasts < 30%), and by EMA for AML patients aged ≥ 65 years not candidates for standard induction chemotherapy, due to its manageable toxicity [50]. Moreover, in a clinical study with ovarian cancer patients there were no differences in the olaparib toxicity between younger and older women [51]. Thus, the favorable safety profile and good oral bioavailability of olaparib makes it a suitable agent to be combined with decitabine for MDS patients ineligible for chemotherapy or BM transplantation. This novel combination may allow dose reduction and overcome some of the limits of hypomethylating agents, by deepening the response and decreasing the burden of treatment-induced cytopenias with the related complications, ultimately leading to improved survival in MDS.

## 4. Materials and Methods

### 4.1. MDS Samples

Freshly isolated primary cells were obtained from BM aspirates of 28 adults with newly diagnosed MDS and one patient with low-blast count AML. Normal BM cells were collected from one hematopoietic stem cell donor and normal CD34+ enriched cells were obtained from the peripheral blood (PB) of three healthy donors following G-CSF-mobilization. MNC were isolated by Lynpholyte-H (Cederlane, Burlington, Canada). In the case of one CD34-enriched mobilized peripheral blood sample, CD34+ cells were purified by the MACS CD34 progenitor isolation kit using immunomagnetic beads (Miltenyi Biotech, Bergisch Gladabach, Germany). All patients and donors gave written informed consent according to institutional guidelines and the study was approved by the local institutional review board (Tor Vergata Hospital, Registry N. 12/16). Routine morphological, immunophenotypic and genetic analysis were carried out at presentation. Conventional karyotyping was performed on BM diagnostic aspirates after short-term culture and analyzed after G-banding.

### 4.2. Cell Culture and Drug Treatment

Freshly isolated BM-MNC were cultured at 37 °C in a humidified atmosphere of 5% CO_2_ for 1 or 2 days before starting the chemosensitivity assays. Briefly, cells were seeded into culture flasks in RPMI medium (Sigma-Aldrich, St. Louis, MO, USA), supplemented with 2 mM L-glutamine (EuroClone, Pero, Milan, Italy), 1% penicillin/streptomycin (Euroclone), 20% fetal bovine serum (FBS) (Gibco, ThermoFisher Scientific, Waltham, MA, USA) and 10 ng/mL of each IL-3, SCF and FLT3LG (PeproTech, Rocky Hill, NJ, USA), which do not have differentiating effects. The OCI-AML2 leukemia cell line (DSMZ-German Collection, Braunschweig, Germany) was cultured in RPMI plus 20% FBS.

Olaparib stock (AstraZeneca, London, UK) was prepared by dissolving 10 mg in 200 µL of DMSO (Sigma-Aldrich) and then diluted with RPMI to the concentration of 2 mM. The final DMSO concentration in the cultures was ≤0.01% (*v*/*v*). The stock solution of decitabine (Cayman Chemical, Ann Arbor, MI, USA) at 2 mM concentration was prepared by dissolving the drug in PBS. Drugs were added at the beginning of the experiments and left in the medium during the entire period of incubation. Since in vitro culture of primary MDS samples could be maintained for 7–10 days only, the assays were performed at up to 7 days after drug exposure. Experiments with normal cells were performed with the same procedure.

For survival assay, cells were seeded in 48 or 24 wells culture plates (10^6^ cells/mL, unless otherwise specified) in duplicate and treated with graded concentrations of olaparib (1–10 μM) or decitabine (0.1–1 μM) for the indicated times. Cells were counted by trypan blue dye exclusion in quadruplicate, and the survival fraction was calculated as proportion of the untreated control. The drug concentration capable of inhibiting 50% of cell growth (IC_50_), compared to untreated control, was calculated with the GraphPad Prism 5 software (San Diego, CA, USA) by using linear regression. The combined effect of olaparib and decitabine was evaluated by the MTS viability test (Promega, Madison, WI, USA), according to the manufacturer’s instructions. Cells were seeded in triplicate (10^4^ cells/mL for OCI-AML2 and 5 × 10^5^ cells/mL for the MDS samples), in 96 wells plates (2 × 10^3^ cells/well and 10^5^ cells/well, respectively) and treated with graded concentrations of olaparib and decitabine. Synergism was assessed by calculating the proportion of cell growth using the CompuSyn software (ComboSyn Incorporated, Paramus, NJ, USA).

### 4.3. Flow Cytometry and Apoptosis Assays

Apoptosis in primary MDS samples was assayed using an annexin-V apoptosis kit (GFP Certified™Apoptosis/Necrosis Detection Kit, Enzo Life Sciences, Farmingdale, NY, USA), according to the manufacturer’s instructions, and analyzed by flow cytometry.

In MDS patients, BM-MNC cells are characterized by a decrease of neutrophil granularity and increased or decreased expression of differentiation antigens or their ratios, although no single specific marker is diagnostic for MDS [30]. Thus, we performed a series of analyses with different antibody combinations to evaluate the maturation pattern induced by olaparib. For immunophenotype analysis, cells were incubated with fluorochrome-tagged monoclonal antibodies anti-CD45 (# 560777), -CD33 (561157), -CD34 (#345804), -CD117 (#339217), -CD123 (#588714), -CD10 (#332776), -CD11b (#333142), -CD15 (#332778), -CD16 (#335035) (BD Biosciences). In particular, we studied the neutrophilic population by analyzing CD11b/CD16 and CD10/CD15 differentiation markers. Samples (5 × 10^4^ cells) were acquired on a multicolor BD FACSCanto II flow cytometer and evaluated using DIVA and FlowJo softwares (BD Biosciences, San Jose, CA, USA).

### 4.4. Immunofluorescence, May-Grunwald Giemsa Staining and FISH Analysis

MDS cells were cytocentrifuged (10^5^ cells), fixed with 4% paraformaldehyde, permeabilized in 0.3% tryton and blocked in 2% bovine serum albumin (Sigma-Aldrich). Slides were incubated with mouse anti-phospho-H2A histone family member X (γH2AX) (#05-636, Millipore, Burlington, MA, USA) followed by goat anti-mouse IgG DyLight 488 (#35502, Thermo Fisher Scientific, Rockford, IL, USA), stained with DAPI (4,6 diamidino-2-phenylindole) and mounted in Fluoromount (Sigma-Aldrich). Analysis was performed using a Leica CTR 6000 fluorescence microscope and LAS AF Lite software (Leica, Wetzlar, Germany).

After 7-day culture, cytospins containing 10^5^ BM-MNC were stained using the May-Grunwald solution. Cells were then observed under a BX61 microscope (Olympus, Tokyo, Japan). and differential counts were performed by two experienced hematologists (A.D.V. and M.T.V.).

Fluorescence in situ hybridization (FISH) was performed on MDS cells cultured for 7 days in the presence of olaparib. Cytogenetic preparations were performed according to the Kreatech FISH probes manufacturer’s instructions (Leica). Slide preparations were hybridized with the Kreatech FISH probes: KBI-2008G, targeting the centromere of chromosome 8, and KBI-10202 targeting chromosome 7q22/7q36 (Leica). A minimum of 200 cells were scored for each preparation by two experienced operators (P.C. and M.G.). Evaluation of the FISH signals was performed using the BX61 Olympus fluorescence microscope.

### 4.5. mRNA and Protein Expression

Total RNA was isolated by Trizol reagent (Invitrogen, Carlsbad, CA, USA). One µg of total RNA was reverse-transcribed (reagents from Applied Biosystem, Warrington, UK) and quantitative real-time PCR (qRT-PCR) was carried out using iQ SYBR Green Supermix, Bio-Rad (BioRad, Hercules, CA, USA). The sequence of the primers used is as follows: PU.1, FW 5′-CAGGGGATCTGACCGACTC-3′ and RV 5′-GCACCAGGTCTTCTGATGG-3′; CEBPA, FW 5′-TTGTTTGTACTGTATGCCTTC-3′ and RV 5′-GCCAGATACAAGTGTTGATAT-3′; GAPDH, FW 5′-CAGCCGAGCCACATCG-3′ and RV5′-TGAGGCTGTTGTCATACTTCTC-3′. A melting curve (62–95 °C) was generated at the end of each run to verify specificity of the reactions. Analysis was performed in triplicate on an ABI-7300 instrument (Applied Biosystems). The 2-ΔΔCt relative quantification method was used to calculate mRNA expression and RNA from untreated cells was used as calibrator. Total proteins were extracted from MDS cells as previously described [16] Immunoblot analysis was performed using anti-CEBPA (sc-166258, Santa Cruz Biotechnology, CA, USA), anti-PU.1 (PA5-17505, Thermo Fisher Scientific), and anti-GAPDH (#2118, Cell Signaling Technology, Danvers, MA, USA) antibodies. Horseradish peroxidase-conjugated IgGs were used as secondary antibodies. The autoradiograms were analyzed by densitometric analysis by ImageJ 1.45s software (National Institutes of Health, Bethesda, MD, USA).

### 4.6. Statistical Analysis

Statistical analysis was performed using the GraphPad Prism 5 software (GraphPad Software, San Diego, CA, USA) and data were reported as mean ± SD (unless otherwise specified). Correlations between olaparib IC_50_ values and proliferation indexes of MDS samples or the prognostic indexes listed in Table 1 were examined using the non-parametric Spearman’s rank test. Differences between two groups were analyzed by the non-parametric Mann-Whitney and Wilcoxon (for matched samples) tests. For multiple comparisons, the non-parametric Kruskal-Wallis or Friedman tests followed by Dunn’s post hoc test were used, as specified in the figure legends. All statistical tests were two-sided. *p* values below 0.05 were considered statistically significant.

## 5. Conclusions

Overall, our data demonstrate that single-agent olaparib, at clinically achievable concentrations, induces cell death of primary MDS cells, while sparing lymphocytes, which are not part of the MDS clone. Furthermore, in this short-term culture model, olaparib favors maturation of myeloid cells towards the neutrophilic lineage, as shown by induction of myeloid-specific transcription factors and increase of the metamyelocyte and neutrophil percentages. Our preclinical data, together with the acceptable toxicity profile of olaparib in vivo, an issue particularly relevant to the elderly MDS population, support the design of clinical trials with PARPi monotherapy or in combination with hypomethylating agents for the management of MDS patients.

## Figures and Tables

**Table 1:** Patient Characteristics.

Prognostic Indexes	Total (*n* = 28)
**Diagnosis**	
MDS-SLD	6
MDS-MLD	11
MDS-EB-1	9
MDS-EB-2	2
**Age (years)** median (range)	73 (58–93)
**Sex** (F/M)	13/15
**Karyotype**	
Normal	18
-Y	1
Trisomy 8	3
5q-	2
20q-	2
2 anomalies *	1
Complex **	1
**BM-blasts** (%) median (range)	4 (3–13)
**Hb (g/dL)** median (range)	11.3 (7.5–16.0)
**WBC (10^9^/L)** median (range)	3.8 (2.0–13.7)
**Neutrophils (10^9^/L)** median (range)	2.8 (0.4–13.1)
**Platelets (10^9^/L)** median (range)	108 (6–608)
**IPSS-R**	
Very-low	0
Low	17
Intermediate	8
High	2
Very-high	1

MDS-SLD: MDS with single-lineage dysplasia; MDS-MLD: MDS with multiple-lineage dysplasia; MDS-EB: MDS with excess of blasts in BM, MDS-EB-1: 5 to 9% blasts, MDS-EB-2: 10 to 19% blasts. * inv,ins(11;9); ** 7q-,12p-,22q-. IPSS-R: Revised International Prognostic Scoring Systems.

## Supplementary Materials

The following are available online at https://www.mdpi.com/2072-6694/11/9/1373/s1, Figure S1: Growth curves of untreated and olaparib treated primary MDS and normal progenitor cell cultures, Figure S2: Analysis of apoptosis in the myeloid and lymphocytic populations of MDS samples, Figure S3: Olaparib induces a decrease in the proportion of CD34+ cells in primary MDS samples, Figure S4: Olaparib induces myeloid differentiation in a primary low blast count AML sample, Figure S5: Olaparib and decitabine combination synergistically impairs survival in the OCI-AML2 leukemia cell line.
