# Supplementary material for: Cytotoxicity and Differentiating Effect of the Poly(ADP-Ribose) Polymerase Inhibitor Olaparib in Myelodysplastic Syndromes
Source: Cancers (Basel). 2019 Sep 16;11(9):1373. doi: 10.3390/cancers11091373 (PMC6769925; doi:10.3390/cancers11091373)
Supplement: Supplementary file 1 [file cancers-11-01373-s001.pdf]

Supplementary Materials: Cytotoxicity and Differentiating Effect of the Poly(ADP-ribose) Polymerase Inhibitor Olaparib in Myelodysplastic Syndromes

Isabella Faraoni, Maria Irno Consalvo, Francesca Aloisio, Emiliano Fabiani, Manuela Giansanti, Francesca Di Cristino, Giulia Falconi, Lucio Tentori, Ambra Di Veroli, Paola Curzi, Luca Maurillo, Pasquale Niscola, Francesco Lo-Coco, Grazia Graziani and Maria Teresa Voso

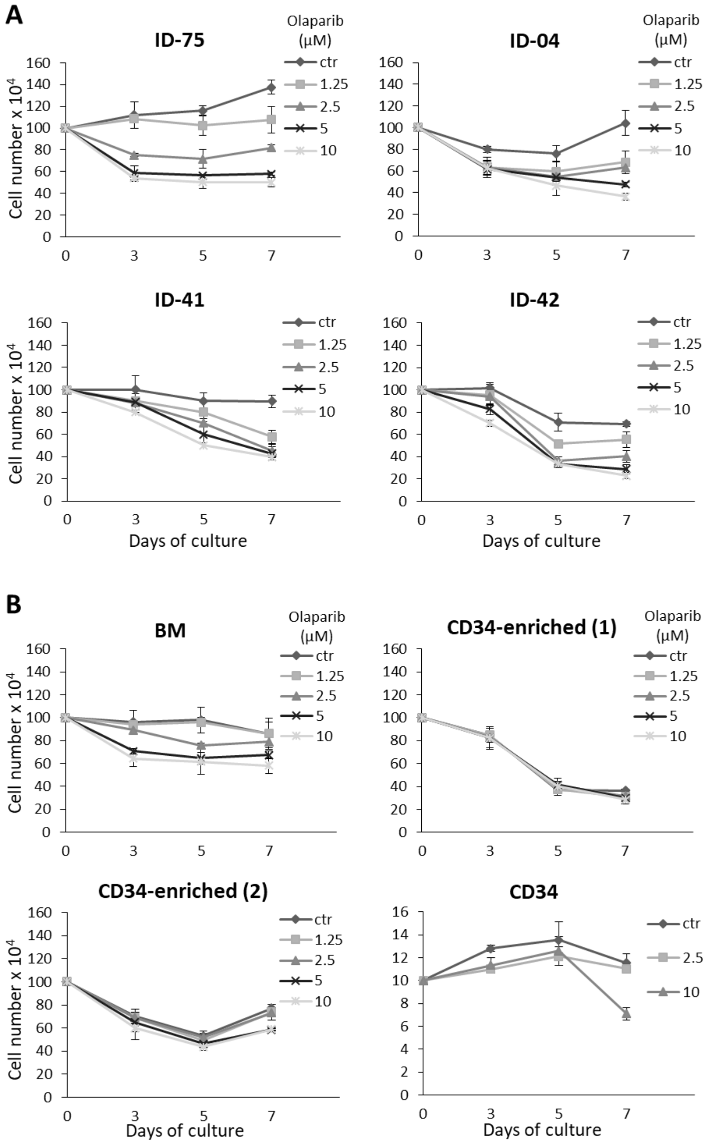

Figure S1. Growth curves of untreated and olaparib-treated primary MDS and normal progenitor cell cultures. (**A**)Bone marrow samples collected from 4 MDS patients were treated with increasing concentrations of olaparib and cultured for 7 days. The indicated MDS samples were characterized by comparable olaparib IC_{50} values (ID-75, ID-04, ID-41 and ID-42 olaparib IC_{50}s: 4.2, 3.9, 3.8 and 4 μM, respectively), but different proliferation rates. (**B**) BM, CD34-enriched mobilized peripheral blood samples and purified CD34+ cells were obtained from healthy donors and exposed to the indicated concentrations of olaparib for 7 days. BM, CD34-enriched (1) and (2), purified CD34+ cells olaparib IC_{50}s: 20.8, 27.0, 26.1, 18.5 μM, respectively. Bars = SD.

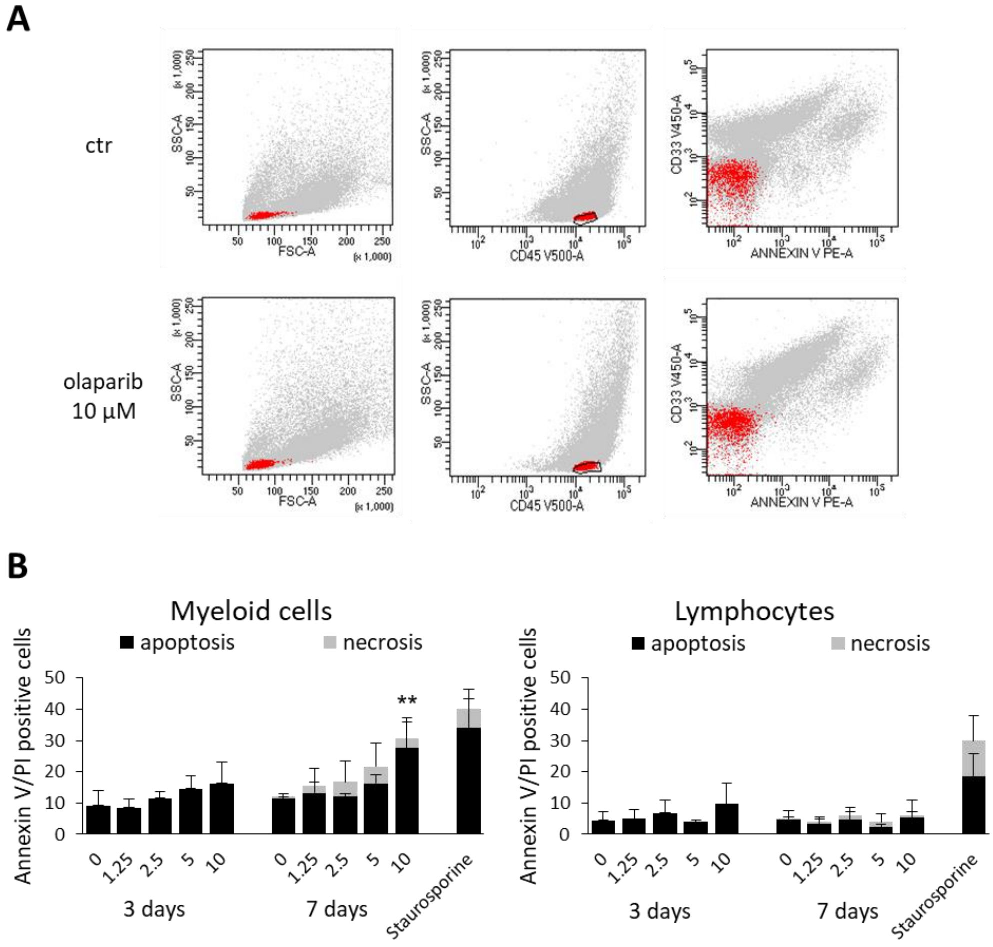

Figure S2. Analysis of apoptosis in the myeloid and lymphocytic populations of MDS samples. (**A**) Representative flow cytometry analysis of one MDS sample (ID-04), untreated or treated with olaparib for 7 days. Dot plots show SSC vs FSC (left panels), side scatter (SSC) vs CD45 staining (middle panels) and CD33 vs annexin V staining (right panels). Lymphocytes, identified as CD45+ and CD33- cells (marked in red), were negative for annexin V. (**B**) Histograms indicate the percentage of apoptotic (Annexin V positive/PI negative) and necrotic (Annexin V positive/PI positive) cells detected in ID-17 MDS samples after exposure to graded concentrations of olaparib for 3 and 7 days, compared to untreated cells. Results are the mean ±SD of three independent experiments. Statistical analysis was performed by Kruskal-Wallis followed by Dunn's test. ** $p < 0.01$, percentage of apoptotic cells vs untreated control.

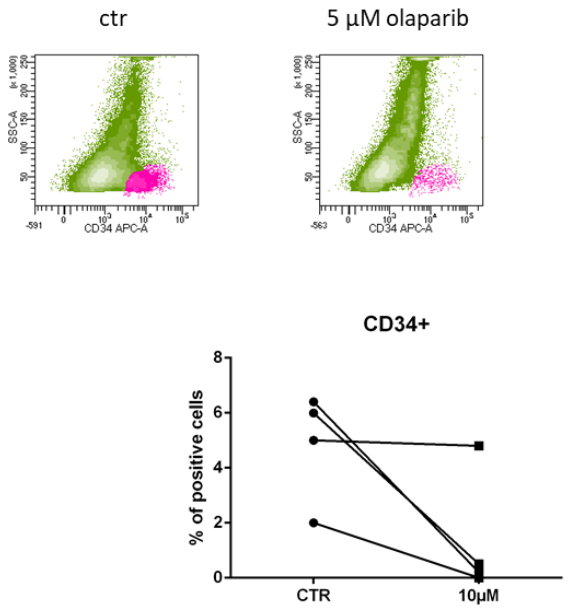

Figure S3. Olaparib induces a decrease in the proportion of CD34+ cells in primary MDS samples. Flow cytometry analysis of CD45+ myeloid cells in MDS samples (ID-02, ID-82, ID-88 and ID-90) untreated or treated with olaparib for 7 days. Representative plots showing the change in CD34+ cells in one MDS sample (ID-88) after treatment with 5 or 10 µM olaparib. The graph indicates that olaparib decreases the percentage of CD34+ immature progenitors in 3 out of 4 MDS samples, as compared to the untreated control.

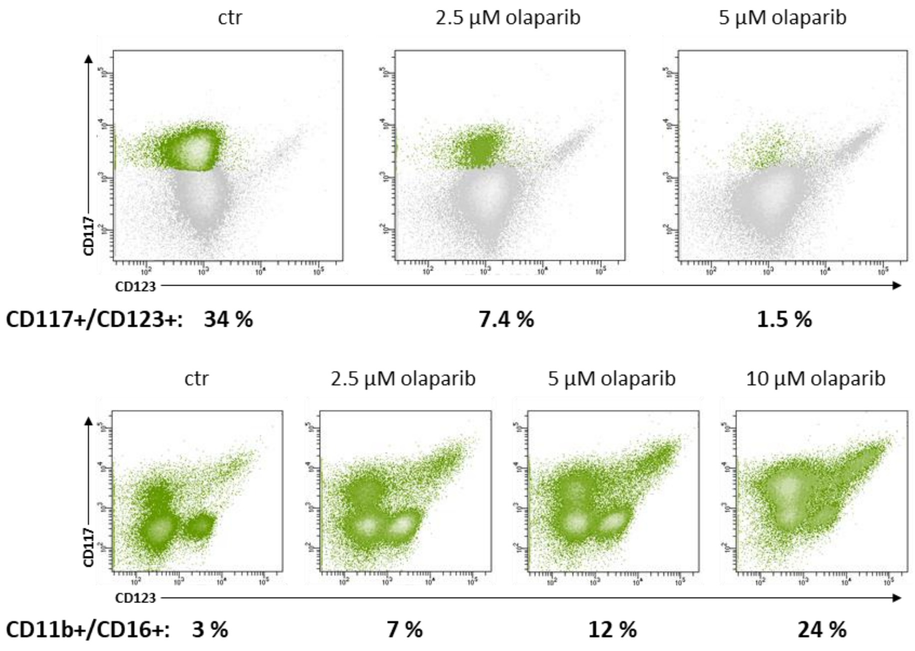

Figure S4. Olaparib induces myeloid differentiation in a primary low blast count AML sample. Flow cytometry analysis of AML CD45+ myeloid cells (BM-blasts: 21% at diagnosis) untreated or treated with olaparib for 7 days (IC_{50} = 1.6 µM). Numbers below plots indicate the percentage of CD117+/CD123+ (upper plots) and CD11+/CD16+ (lower plots) cells. Olaparib induces a dose-dependent decrease of CD117+/CD123+ immature progenitors and an increase of CD11b+/CD16+ mature neutrophils.

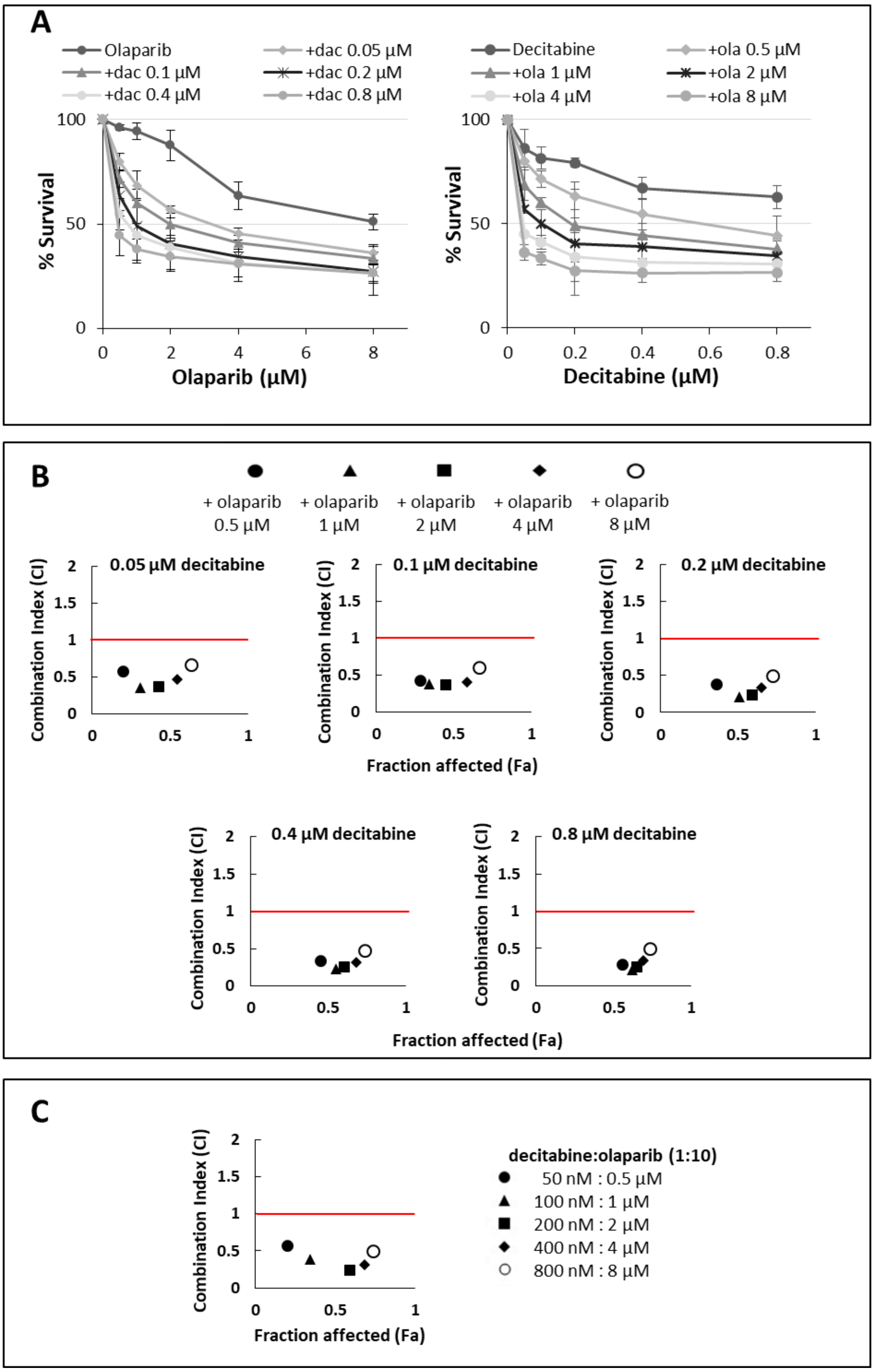

Figure S5. Olaparib and decitabine combination synergistically impairs survival in the OCI-AML2 leukemia cell line. The OCI-AML2 cell line was treated with olaparib (0–8 µM), decitabine (0–0.8 µM) or with the indicated combination at time 0. After 72 h of culture, cells were analyzed by the MTS assay. (**A**) Percentage of surviving cells after treatment with increasing concentrations of olaparib, as single agent and in combination with the indicated concentrations of decitabine (dac) (left panel), or with increasing concentrations of decitabine as single agent and in combination with the indicated concentrations of olaparib (ola) (right panel). Values are the mean ±SD of three independent experiments. (**B**) Plots show the fraction of affected cells (Fa, *x* axis) and the combination index (CI, *y* axis) as assessed by the CompuSyn model. Data refer to OCI-AML2 cells treated with a fixed concentration of decitabine (indicated in the plot legend) in combination with increasing olaparib

concentrations. (**C**) Plot shows the results obtained with a fixed drug ratio (1:10) corresponding to equitoxic drug concentrations. In OCI-AML2 cells, olaparib and decitabine IC_{50} values were 8.8 ± 1.0 μM and 0.9 ± 0.1 μM, respectively. CI < 1, synergistic activity; CI = 1, additive effect; CI > 1, antagonism.

© 2019 by the authors. Licensee MDPI, Basel, Switzerland. This article is an open access article distributed under the terms and conditions of the Creative Commons Attribution (CC BY) license (http://creativecommons.org/licenses/by/4.0/).
